# *In vitro* Effectiveness of Commercial Bacteriophage Cocktails on Diverse Extended-Spectrum Beta-Lactamase Producing *Escherichia coli* Strains

**DOI:** 10.3389/fmicb.2016.01761

**Published:** 2016-11-03

**Authors:** Aycan Gundogdu, Darajen Bolkvadze, Huseyin Kilic

**Affiliations:** ^1^Department of Microbiology and Clinical Microbiology, Faculty of Medicine, Erciyes UniversityKayseri, Turkey; ^2^Genome and Stem Cell Center (GENKOK), Erciyes UniversityKayseri, Turkey; ^3^Laboratory of Molecular Biology, G. Eliava Institute of Bacteriophages, Microbiology and VirologyTbilisi, Georgia

**Keywords:** ESBL-*E. coli*, bacteriophage, antibiotic resistance, phage therapy, phylogenetic grouping

## Abstract

The objective of this study is to determine the *in vitro* susceptibility of Georgian bacteriophage cocktails on multidrug resistant (MDR) extended-spectrum beta-lactamase producing *Escherichia coli* (ESBL-EC) isolated from patients’ blood and urine cultures. A total of 615 *E. coli* isolates were included in this study. Phene Plate (PhP)-typing and phylogenetic grouping were used for the typing. Antimicrobial resistance profiles and ESBL production of all isolates were confirmed according to Clinical and Laboratory Standards Institute (CLSI) criteria. The activities of four bacteriophage cocktails (Enko-phage, SES-bacteriophage, Pyo-bacteriophage, and Intesti-bacteriophage) were determined against 142 ESBL-EC using *in vitro* spot tests. According to this, Enko-phage were active against 87.3% of the tested strains while that ratio was 81.7% for Intesti-bacteriophage, 81.7% for Pyo-bacteriophage, and 59.2% for SES-bacteriophage cocktails. Based on the contingency tests, the phage cocktails were observed to be statistically significantly (*p* < 0.001) more effective on ESBL-EC strains belonging to phylogenetic groups D and B2. The employed phage cocktails were found to be affective against all tested resistant types. These results are promising especially for the infections that are caused by MDR pathogens that are difficult to treat. As this is a preliminary step to the potential clinical trials to be designed for the country, *in vitro* confirmation of their success on a MDR ESBL-EC collection should be accepted as an initial action, which is encouraging to consider clinical trials of phage therapy especially in countries which are not introduce phage therapy.

## Introduction

*Escherichia coli* is the leading bacterial pathogens responsible for intestinal and extraintestinal infections, including urinary tract infections, bacteremia, and meningitis (Kaper et al., 2004). Certain strains of *E. coli* have been shown to carry certain genes coding for enzymes that hydrolyze a wide range of beta-lactams and are referred to as extended-spectrum beta-lactamase producing *E. coli* (ESBL-EC). In recent years, the dissemination of ESBL-EC has become a serious health problem worldwide including Turkey (Pitout and Laupland, 2008; Rodriguez-Bano and Pascual, 2008; Tasbakan-Isikgoz et al., 2011; Hawser et al., 2012). ESBL producing pathogens are known to be resistant not only first, second, and third generation cephalosporins and monobactams, but also to several other classes of antibiotics. As a result, infections that are difficult to treat occur, and occasionally the use of “antibiotics of the last resort” such as carbapenems, colistin is inevitable (Cantón et al., 2012). However, unfortunately, emergence of resistance against these drugs of last resort is also observed around the world (Schwaber and Carmeli, 2008; Hasman et al., 2015). The upcoming threat of increase in the prevalence of untreatable infections has motivated the quest for alternatives of antibiotic therapy.

Bacteriophages (phages) are natural parasites of bacteria and they have been considered as effective agents for the treatment of bacterial infections since 1920s (Sulakvelidze et al., 2001; D’Herelle, 2007). The reason phages have been used as antimicrobial agents in that they can recognize, bind onto, and reproduce within a bacterial host, resulting in rapid cell lysis. However, poor understanding of phage biology and inconsistent outcomes experienced in early trials resulted in the phage therapy never being adopted universally (Sulakvelidze et al., 2001; Kutter et al., 2010). The advancement and consequently widespread use of antibiotics left phage therapy remaining as a common practice confined in the former Iron Curtain Countries where it has been successfully applied for around 90 years. Currently, although antibiotic therapy is successful in the treatment of majority of infections, frontline therapies are not effective in noticeable amount exceptions (Gould, 2009; Gootz, 2010; Keske et al., 2014) which is the underlying reason for the revitalization of phage therapy in bacterial infections around the world (Parracho et al., 2012). Thus, adopting this renewed approach we designed this preliminary study on ESBL-EC since it is a significant infection control and treatment challenge in the world.

This study aimed to examine the *in vitro* activity of Georgian bacteriophage cocktails which are used as part of standard clinical practice in the Republic of Georgia on a well-characterized ESBL-EC isolated from Turkish patients’ blood and urine. As this is a first step to the potential clinical trials to be designed for the countries that have reached a very high prevalence country wide, the study is of capital importance.

## Materials and Methods

### Sample Collection

Clinical samples belonging to five experimental groups were included in this study. Experimental groups are (i) the blood of hospitalized patients, (ii) the urine of hospitalized adult patients, (iii) the urine of hospitalized pediatric patients, (iv) the urine of adult outpatients, and (v) the urine of pediatric outpatients. All samples are collected from the patients attending a 1300-bed tertiary hospital in Kayseri, Turkey, between 2014 and 2016. The samples did not include descriptive information from any patients. Identification of *E. coli* strains was performed using conventional microbiological methods and VITEK-2 (bioMérieux) automated system. Extraction of chromosomal DNA was done by growing a single colony of the isolates in Luria–Bertani (LB) broth overnight, collecting pellet in 100 ml sterile Milli-Q water, and boiling at 95°C for 15 min. *E. coli* strains were subjected to confirmatory tests using PCR amplification of the universal stress protein (*uspA*) gene as previously described by Chen and Griffiths (1998).

### Typing of Isolates

#### Phene Plate (PhP) Fingerprinting

All strains were typed using a high-resolution biochemical fingerprinting method specifically developed for *E. coli* strains (PhPlate AB, Stockholm, Sweden; Landgren et al., 2005). Inoculation of the plates was done according to the manual instruction and the plates were incubated at 37°C. The rate of each reaction was evaluated by measuring the absorbance value in each well after 7, 24, and 48 h of incubation using a digital scanner. After the final scan, the PhPlate software was used to create absorbance (A620) data from the scanned plate images and the mean absorbance in each well over the three readings was calculated, yielding the biochemical fingerprint for each isolate. The biochemical fingerprints of the isolates were compared pairwise and the similarities among the isolates were calculated by the correlation (similarity) coefficient and clustered according to the unweighted pair group method with arithmetic averages (Sneath and Sokal, 1973; Saeedi et al., 2005). Isolate pairs having a similarity coefficient above the default identity level (0.975) of software was regarded as identical and assigned to the same biochemical phenotype. All data handling, including calculations of correlations and coefficients as well as clustering were performed using the PhPlate software version 4002 (PhPlate AB, Stockholm, Sweden).

#### Phylogenetic Grouping

Phylogenetic grouping (A, B1, B2, D) was done for ESBL-EC strains using multiplex PCR with primers coding for *chuA* and *yjaA* genes and the DNA fragment TSPE4.C2 according to Clermont et al. (2000).

Strains belonging to the same biochemical phenotype and phylogenetic groups were considered as members of the same clone and were regarded as common types, and those differing by one method were regarded as single types.

### Phenotypic ESBL Detection and Antimicrobial Resistance Test

ESBL production of the isolates was screened by the double-disk synergy on Mueller-Hinton agar using cefotaxime, ceftazidime, cefpodoxime, and ceftriaxone with and without clavulanic acid (10 mg), according to Clinical and Laboratory Standards Institute (CLSI), recommendations (Clinical and Laboratory Standards Institute [CLSI], 2015). After phenotypic confirmation, the susceptibility of ESBL-EC producing strains was tested against nine antimicrobial agents using Kirby–Bauer disk diffusion methods according to CLSI guidelines. Antimicrobial agents included were ampicillin/sulbactam, ciprofloxacin, gentamicin, sulfamethoxazole, amoxicillin/clavulanate, piperacillin/tazobactam, amikacin, cefepime, imipenem, and ertapenem. *E. coli* ATCC 25922 and ESBL-positive *bla_ctx-m-15_* ESBL-producing strains were used as quality control strains.

### *In vitro* Susceptibilities of Bacteriophage Cocktails

Among all ESBL-EC, one representative from each PhP-common type and all single types are included in phage assay. Spot tests were performed to determine the activities of four commercially available bacteriophage cocktails on each non-replicating ESBL-EC isolate. The bacteriophage cocktails produced in the George Eliava Institute and they were commercially available in sterile media containing 1 × 10^5-6^ phage particles in 1 mL for Pyo-bacteriophage and Intesti-bacteriophage in Georgia. For the remaining two cocktails, the plaque forming unit/mL concentration is not available.

For spot test, all ESBL-EC isolates were initially tested for their purity on MacConkey agar no. 3. One single colony were transferred onto LB agar and incubated at 37° for 18 h. These steps were repeated twice to yield desired colonies (i.e., round shaped) with similar morphology in the agar plates. Single colonies were picked and incubated in LB broth for about 3 h. Then, 100 μL of the bacterial culture (A600 ca. 0.5) mixed with 5 ml of the molten semi-solid LB agar (0.6%) and over laid on the surface of the LB agar. After 10 min, 10 μL of phage suspension was applied on the bacterial layer and incubated at 37°C overnight. Bacterial sensitivity to phage preparations was established by lysis at the spot where the phage was deposited (Gabrilovich, 1973). Observing confluent, semi-confluent, opaque lysis or individual plaques determined the isolate susceptibility. When the lysis was not possible, the corresponding isolates were determined to be resistant. Each test was repeated thrice.

The following four commercial phage cocktails were included in this study.

Enko-phage active against *Salmonella typhimurium*, *Salmonella enteritidis*, *Salmonella heidelberg*, *Salmonella newport*, *Salmonella cholerae*, *Salmonella oranienburg*, *Salmonella dublin*, and *Salmonella anatum*; Shigella flexneri (serovars 1, 2, 3, 4) and Shigella sonnei (six different serovars), enteropathogenic E. coli, *Staphylococcus aureus*, *Staphylococcus epidermidis*, and *Staphylococcus saprophyticus*.

SES-bacteriophage active against Staphylococci (*S. aureus*, *S. epidermidis*, and *S. saprophyticus*); Streptococci (*Streptococcus pyogenes*, *Streptococcus sanguis*, *Streptococcus salivarius*, and *Streptococcus agalactiae*) and different serotypes of enteropathogenic *E. coli* serovars.

Pyo-bacteriophage active against *Staphylococcus* spp., *Streptococcus* spp, *Pseudomonas aeruginosa*, *Proteus mirabilis*, *Proteus vulgaris*, and *E. coli*.

Intesti-bacteriophage active against *Shigella* spp., *Salmonella* spp., *E. coli*, *Staphylococcus* spp., *Enterococcus* spp., *Proteus* spp., *P. aeruginosa*.

## Results

A total of 615 *E. coli* were isolated from urine (*n* = 433) and blood (*n* = 182) cultures of patients (one isolate per patient) included in each experimental group (**Table 1**). One hundred and seventy-five out of 615 *E. coli* strains were confirmed to be genuine ESBL producers. All 175 ESBL-EC isolates were grouped into 20 common types (i.e., types with more than two identical isolates) and 122 single types based on typing methods. The lowest number of identical isolates in a common type was 2, and the highest number of identical isolates belonging to a common type was 5. With the consideration of clonality the number of representative isolates was determined to be 142 for ESBL-EC. Phylogenetic distribution of these 142 strains revealed that, 40.8% of the isolates belonged to group D, 38.1% belonged to B2, 14.2% belonged to group A, and 7% belonged to B1.

**Table 1:** Numbers of confirmed *E. coli* strains included in this study within each experimental group.

Experimental groups	Number of *E. coli* strains		
	ESBL -ve	ESBL +ve	Total
**Urine**			
Hospitalized adults	74	43	117
Hospitalized children	26	19	45
Adult outpatients	92	26	118
Children outpatients	131	22	153
**Blood (adults and children)**	117	65	182
**Total**	440	175	615

*ESBL -ve, strains not producing extended-spectrum beta-lactamases; ESBL +ve, strains producing extended-spectrum beta-lactamases.*

The rates of ESBL-EC within each experimental group were found to be ranging between 14.7% (for urine culture of children outpatients) and 40.4% (for blood culture of hospitalized patients) (**Table 2**). In addition to ESBL production all isolates were found to be multidrug resistant (MDR, resistant to >3 classes; Magiorakos et al., 2012; **Table 3**). The rate of Carbapenem non-susceptible (non-S) strains was found to be ranging between 2.2 and 5.1% among different experimental groups of ESBL-EC.

**Table 2:** Distribution of 615 *E. coli* strains within each experimental group after PhP-typing.

	Number of representative strains				PhP-types		
Experimental groups	ESBL -ve	ESBL +ve	Total	%ESBL +ve	Common types	Single types	DI
**Urine**							
Hospitalized adults	65	33	98	33.7	12 (31)	86	0.996
Hospitalized children	23	14	37	37.9	5 (13)	32	0.991
Adult outpatients	70	21	91	23.01	18 (45)	73	0.994
Children outpatients	87	15	102	14.07	25 (76)	77	0.990
**Blood**	87	59	146	40.4	23 (59)	123	0.996

*ESBL -ve, strains not producing extended-spectrum beta-lactamases; ESBL +ve, strains producing extended-spectrum beta-lactamases; DI, diversity index. The numbers in the bracket indicated how many strains belong to common types.*

**Table 3:** Antimicrobial resistance percentages of 142 ESBL-EC strains.

Experimental groups	Antimicrobial agents tested									
	SAM	CIP	GEN	STX	AMC	TZP	AMK^∗^	FEP	IMI	ERT
**Urine (83)**										
Hospitalized patients (47)	82	73.3	42.3	74.5	51.2	30	5	100	2.2	2.2
Outpatients (*n* = 36)	79.2	60.5	24.4	60.2	47.6	20	0	100	0	0
**Blood (*n* = 59)**	95	67.8	48	78	59.5	40.7	3.4	96.6	5.1	3.4

*SAM, ampicillin/sulbactam; CIP, ciprofloxacin; GEN, gentamicin; STX, sulfamethoxazole; AMC, amoxicillin-clavulanate; TZP, piperacillin/tazobactam; AMK, amikacin; FEP, cefepime; IMI, imipenem; ERT, ertapenem. ^∗^Reduce susceptibility.*

All 142 non-replicating ESBL-EC were included in the bacteriophage assay. According to phage susceptibility assay 131 (92.3%) of the ESBL-EC strains were determined to be susceptible to at least one phage cocktail. Among those, Enko-phage, Intesti- bacteriophage, and Pyo-bacteriophage preparations were active against greater number of isolates than SES-bacteriophage (87.3, 81.7, 81.7, and 59.2%, respectively). Resistance and sensitivity profiles of these four phage preparations are given in **Table 4**.

**Table 4:** Sensitivity profiles of tested phage cocktails on 142 ESBL-EC included in this study.

	Number of ESBL-EC strains			
	ENKO	SES	PYO	INTESTI
Confluent	38	1	38	35
Semi-confluent	29	10	27	20
Opaque lysis	23	7	23	27
Individual plaques	34	66	28	34
Resistant	18	58	26	26

*ENKO, Enko-phage; SES, SES-bacteriophage; PYO, Pyo-bacteriophage; INTESTI, Intesti-bacteriophage.*

Considering the phage susceptibility distributions of the tested strains with respect to different phylogenetic groups, it was observed that each phylogenetic group shows different phage susceptibility characteristics. While Enko, Pyo, and Intesti phages are dominantly effective on B2 and D phylogenetic groups (7.4% resistance rate for all phage cocktails in B2, 3.4, 5.2, and 8.6% resistance rates for Enko, Pyo, and Intesti, respectively in D), a more even susceptibility response can be observed for A and B1 phylogenetic groups (*p* < 0.00001, chi-square test). This trend can also be observed for SES cocktail with statistical significance (*p* = 0.000295, chi-square test), yet effectiveness for B2 and D phylogenetic groups seems to be less than the other cocktails (27.7 and 34.4% resistance rates for B2 and D groups, respectively) (**Table 5**).

**Table 5:** Association between phylogenetic groups and phage resistance among 142 ESBL-EC strains.

	Number of strains	Number of strains in each phylogenetic groups				
		B2	D	A	B1	*p* value
**ENKO**						
Resistant	18	4	2	10	2	<0.00001
Susceptible	124	50	56	10	8	
**SES**						
Resistant	58	15	20	17	6	0.000295
Susceptible	84	39	38	3	4	
**PYO**						
Resistant	26	4	3	14	5	<0.00001
Susceptible	116	50	55	6	5	
**INTESTI**						
Resistant	26	4	5	13	5	<0.00001
Susceptible	116	50	53	7	5	

*ENKO, Enko-phage; SES, SES-bacteriophage; PYO, Pyo-bacteriophage; INTESTI, Intesti-bacteriophage; p values were calculated using chi-square test.*

ESBL-EC strains isolated from hospitalized patients were more susceptible to Enko-phage than other phage preparations, whereas outpatients’ isolates were more susceptible to Pyo-bacteriophage than the other cocktails. Eleven (7.7%) of the ESBL-EC isolates were found to be resistant to all commercially available bacteriophage cocktails. These 11 ESBL-EC were isolated from blood (*n* = 5), from the urine of hospitalized adults (*n* = 2), from the urine of pediatric outpatients (*n* = 2) and from the urine of adult outpatients (*n* = 2). The susceptibility/non-susceptibility patterns of *E. coli* showed no correlation with their antibiotic resistance patterns.

## Discussion

Over the past decade, the prevalence of ESBL-EC has attracted the attention of health authorities due to their increasing rates on a daily basis (Paterson and Bonomo, 2005). Recent studies have shown that the prevalence of ESBL-EC in different countries can be as high as 63%, with the highest rate belonging to *E. coli* isolated from ICU patients in Turkey (Hawser et al., 2009; Magiorakos et al., 2012; Nakai et al., 2016). Reports indicated that ESBL-EC rates are generally very high in Turkey; however, previous studies generally do not take the clonal relationships of the isolates into account (Kacmaz et al., 2005). Although molecular typing methods such as pulsed field gel electrophoresis (PFGE) or multilocus sequence typing (MLST) have been used extensively for determining clonal relationships of the investigated strains, for practical purposes, we employed the PhP-typing method that specifically developed to discriminate *E. coli* strains (Kühn et al., 1995). This system has been used in several epidemiological studies (Ahmed et al., 2006; Cassanovas-Massana and Blanch, 2013) and was shown to have similar discriminatory power with the genotypic methods (Ansaruzzaman et al., 2000; Vollmerhausen et al., 2011). Thus, the ESBL-EC rates (between 14.7 and 40.4%) reported in this study might be indicating a more accurate estimate for Turkish outpatients and hospitalized patients. However, the ESBL-EC ratio especially for hospitalized patients, are still very high and the required precautions should be taken urgently. In addition to ESBL production, the high antibiotic resistance rates found this study might be attributed to their frequent preference in empirical treatment and the overall antibiotics usage policies. A previous Turkish meta-study highlighted the increase of ciprofloxacin, cefepime, co-trimoxazole resistance and ESBL production during the study period of 1996–2012 (Aykan and Cifci, 2013). As co-resistance to aminoglycosides and quinolones is developed quickly during the ESBL-EC treatments, carbapenems have been the drugs of choice when an infection is caused by ESBL-EC. In this study carbapenem resistance ratio was found to be between 2.2 and 5.1%. Since the carbapenems are still active against the majority of ESBL-EC, clinicians increasingly prefer carbapenems for empiric or definitive therapy in community-onset and nosocomial infections caused by this pathogen (Rodríguez-Bano et al., 2012). However, this situation results in a frequent explosion of carbapenems to bacteria, which consequently makes the spreading of carbapenemase-producing bacteria scenario more probable and worrisome.

The treatment difficulties on infections caused by MDR pathogens continue to be a vexing problem. Thus, alternative approaches as the treatment options are in quest. Being one of the most popular approaches employing phages as anti-infective agents was adopted in this study. The results of the study indicate that, a great majority (131/142, 92.3%) of diverse ESBL-EC strains were susceptible to at least one phage cocktail. Furthermore, considering the high diversity among each experimental groups based on typing methods, it might be concluded that phages are effective on a broad range of ESBL-EC clones. This is quite promising especially from the point of infections difficult to treat that are caused by MDR pathogens. In an Irish study conducted by Fitzgerald-Hughes et al. (2014), 82% of ESBL-EC isolated from patients were found to be susceptible to the same bacteriophage cocktails used in current study. Higher percentage found in our study might be explained by the geographical contact between Georgia and Turkey. Because of the neighboring geographies, residents go back and forth between these two countries resulting in bacterial transfers. Thus, as the producer (The G. Eliava Institute of Bacteriophages, Microbiology and Virology) updates its commercial phages regularly, new phages against new strains disseminated from Turkey (e.g., via patients, food, etc.) might find a room in these bacteriophage cocktails. However, in another study conducted by Sybesma et al. (2016) in which they used 29 *E. coli* bacteriophages from the Eliava collection in addition to four commercial phage cocktails on *E. coli* set compromising ESBL negative and positive *E. coli*, the phage susceptibility rate was found to be 92.6%. In the current study, 7.7% ESBL-EC isolates were found to be resistant to all bacteriophage cocktails tested. Since the phage cocktails are originated from Georgian biogeography, the phages of these strains might not be contained in the cocktail preparations. On the other hand, isolation of bacteriophages, which are able to be effective on these resistant strains from those pathogens’ own environment might be possible. With a specified preparation of phage cocktails and/or with the adaptation of bacteriophages (Sybesma et al., 2016), even more successful susceptibility results are potentially achievable. In our study, SES-bacteriophage was the least active (59.2%) cocktail whereas in Fitzgerald-Hughes et al.’s (2014) study, SES-bacteriophage was the most active phage preparation. This outcome might be the indication of different *E. coli* sub-groups being common in Ireland and in Turkey. According to our study, bacteriophage cocktails were found to be effective against all antimicrobial resistance types tested, including carbapenem resistance. This orthogonality between the phage activity and the antimicrobial resistance might be an encouraging factor for the adoption of phage therapy as an alternative or complementary option to difficult to treat infections. For instance, in this study the majority of ESBL-EC (58.4%) were isolated from the urine cultures of the patients. For these patients, carbapenems are generally considered as the drug of choice (Prakash et al., 2009) and in Turkey there are no oral options for carbapenem treatment. Therefore, treatment for the urinary tract infections caused by ESBL-EC generally requires hospitalization due to intravenous antibiotic therapy application. This is an unfavorable situation as it increases the odds of the patients’ being exposed to nosocomial infections, as well as increasing the medical expenses. Considering these multiple drug resistant pathogens are frequently susceptible to the tested phages according to our *in vitro* experiments, employing phage therapy (oral) with *in vitro* tested phage preparations could be an alternative or complementary approach for the cases that narrow spectrum antibiotic therapy is not sufficient or hospitalization is required.

The association of the phylogenetic groups with the virulence characteristics of *E. coli* has been repeatedly reported in the literature, which allows a separation of commensal (A and B1) and pathogenic (B2 and D) strains (Bingen et al., 1998; Duriez et al., 2001). As B2 and D are mainly accepted to contain the pathogenic strains, while A and B1 phylogenetic groups are known to mostly include environmental/commensal strains, the altering characteristics might reveal a feature for the employed phage cocktails. According to our results, the cocktails have a particular effectiveness on the pathogenic strains. The underlying reason for that phenomenon might be an artificial selection of “pathogen killing” bacteriophages during phage adaptation procedures since they are selected on clinical strains. Anyhow, this finding can lead us to draw a conclusion that the bacteriophage cocktails are highly specific to selected pathogens, which might turn out to be a preferable feature as they are less likely to harm beneficial components of human microbiome even when they are used orally.

Journals published since 1920s have indicated that the success rate of the *in vivo* phage therapy—orally, rectally, locally, intrapleural injections or intravenously, etc.—is nearly 95%, clinical improvements or full recovery is usually observed within at most 7 days with no reports of serious complications or side effects (allergy, kidney or liver failure, etc.) associated with their use (Chanishvili, 2012). Although clinical practice of phage therapy as a common medical practice has not been approved yet, except a few Eastern European countries, some studies conducted in EU have already reached the clinical trial phase (Merabishvili et al., 2009; Rhoads et al., 2009; Wright et al., 2009; Pelfrene et al., 2016). As an example, a study started in 2013 and conducted within European Union PF7 programme with the collaboration of researchers from Belgium, France, and Switzerland, achieved their first phage therapy application of burn wound infections on human patients in July 2015^1^.

## Conclusion

This study demonstrated the *in vitro* activity of bacteriophage preparations that are used as a part of standard clinical practice in the Republic of Georgia, against MDR ESBL-EC isolated from Turkish patients. Although bacteriophage therapy has been a successful part of standard healthcare practice for decades, the non-practicing camp has been skeptical to accept bacteriophages as alternative anti-infectives. However, the need for novel antimicrobial therapies have always been of great importance, yet with the emergence of pan-resistant Gram-negative pathogens recently (Arias and Murray, 2009; Glupezynski et al., 2010) it could be argued that the quest has never been that crucial since the beginning of the antibiotic era. Thus, *in vitro* confirmation of their success on a well-characterized isolate collection is reported as an initial action, which is encouraging for their clinical use especially for infections with treatment difficulties. Moreover, the effectiveness of generic phage cocktails which are not specifically produced for strains of concern is an implication that the spectrum of phages is large enough to allow their general use.

## Availability of Data and Materials

The datasets analyzed during the current study are available from the corresponding author on reasonable request.

## Author Contributions

AG conceived and designed the study, performed the tests, analyzed and interpreted the data, and wrote the manuscript. HK collected the strains and performed the automated identification and antimicrobial susceptibility tests. DB participated in the phage study and writing and revision of the manuscript. All authors read and approved the final manuscript.

## Conflict of Interest Statement

The authors declare that the research was conducted in the absence of any commercial or financial relationships that could be construed as a potential conflict of interest.

## Footnotes

1 http://www.phagoburn.eu/
